# Fabrication of Iron Pyrite Thin Films and Photovoltaic Devices by Sulfurization in Electrodeposition Method

**DOI:** 10.3390/nano11112844

**Published:** 2021-10-26

**Authors:** Zheng Lu, Hu Zhou, Chao Ye, Shi Chen, Jinyan Ning, Mohammad Abdul Halim, Sardor Burkhanovich Donaev, Shenghao Wang

**Affiliations:** 1 Materials Genome Institute, Shanghai University, Shanghai 200444, China; luzhengf@shu.edu.cn (Z.L.); zhouhushu@163.com (H.Z.); 18852726530@163.com (C.Y.); sdzeg05@163.com (S.C.); 2 Department of Materials Science & Engineering, University of Rajshashi, Rajshahi 6205, Bangladesh; halimtsukuba2012@gmail.com; 3 Faculty of Electronics and Automation, Tashkent State Technical University, University Str. 2, Tashkent 100095, Uzbekistan; sardor.donaev@gmail.com; 4 Energy Materials and Surface Sciences Unit (EMSSU), Okinawa Institute of Science and Technology Graduate University (OIST), 1919-1 Tancha, Onna-son 904-0495, Okinawa, Japan

**Keywords:** iron pyrite, electrochemical deposition, thiourea, sulfurization

## Abstract

Iron pyrite is a cheap, stable, non-toxic, and earth-abundant material that has great potential in the field of photovoltaics. Electrochemical deposition is a low-cost method, which is also suitable for large-scale preparation of iron pyrite solar cells. In this work, we prepared iron pyrite films by electrochemical deposition with thiourea and explored the effect of sulfurization on the synthesis of high-quality iron pyrite films. Upon sulfurization, the amorphous precursor film becomes crystallized iron pyrite film. Optical and electrical characterization show that its band gap is 0.89 eV, and it is an n type semiconductor with a carrier concentration of 3.01 × 10^19^ cm^−3^. The corresponding photovoltaic device shows light response. This work suggests that sulfurization is essential in the electrochemical preparation for fabricating pure iron pyrite films, and therefore for low-cost and large-scale production of iron pyrite solar cells.

## 1. Introduction

Photovoltaic cells are a critical technology in producing green energy and suppressing global warming. The development of photovoltaic technology requires cheap, stable, non-toxic, and earth-abundant materials. Iron pyrite (FeS_2_) is a photovoltaic material that has attracted researchers in recent years [1,2]. It possesses high stability and nontoxicity with an indirect optical band gap of 0.95 eV. Most importantly, it shows a high absorption coefficient of α = 6 × 10^5^ cm^−1^ (for λ < 700 nm), which means that the absorption capacity of 20-nm-thick iron pyrite film is comparable to that of 300-μm-thick crystalline silicon (α ≥ 1.9 × 10^3^ cm^−1^ for λ ≤ 700 nm) [3,4]. However, its development and application have been restricted for decades [5], owing to sulfur vacancies [6], undesired doping [7], surface conduction [8], and so on. So far, the record power conversion efficiency (PCE) of FeS_2_-based solar cells is 2.8% [9,10,11,12,13,14,15]. Therefore, extensive investigation on FeS_2_ is still needed, including material synthesis, defect properties, and device physics.

Researchers have tried various methods to synthesize iron pyrite films, such as hydrothermal, hot injection, spin coating, chemical vapor deposition, physical vapor deposition, spray pyrolysis, and electrochemical deposition (ECD) [1,3,14,16,17]. Among them, ECD is the simplest and most cost-efficient method, and can produce a large-area film without a vacuum [17,18,19]. These merits make it suitable for production on an industrial scale. Sulfurization is proven to be not only crucial to synthesize pure semiconductors, such as CZTS and In_2_S_3_ [20,21,22], but also be essential for improving the crystallinity of spin-coated or sputtered iron pyrite films [7,13]. However, sulfurization has not been utilized as a post-treatment in the synthesis of FeS_2_ film with thiourea based on ECD [23]. Therefore, we suppose that sulfurization may further enhance the film quality of FeS_2_ film prepared by ECD.

In the present work, we fabricated FeS_2_ thin films using the ECD technique, and investigated the effect of sulfurization temperature on the properties of iron pyrite films. The results show sulfurization at 450 °C is very important for forming crystallized, phase-pure, and dense FeS_2_ thin film. With the prepared FeS_2_ thin films, the FeS_2_/P3HT-based solar cell was prepared and it shows photovoltaic property.

## 2. Materials and Methods

Thiourea (Adamas-beta from Shanghai, China, 99%), polyethylene glycol (6000) (General-reagent from Shanghai, China, AR, abbreviated as PEG(6000)), ferrous sulfate heptahydrate (Aladdin, Shanghai, China 99.95%), potassium chloride (Aladdin from Shanghai, China, 99.8%), sulfuric acid (SCR from Shanghai, China, 95.0~98.0%), sulfur powder (SCR from Shanghai, China, ≥99.999%), poly(3-hexylthiophene-2,5-diyl) (p-OLED from Shanghai, China, MW~37000, abbreviated as P3HT), chlorobenzene (Aladdin Shanghai, China, 99.5%), and iron pyrite powder (Hawk from Shanghai, China, 99%) were used in the experiments. Electrochemical deposition was done in an aqueous solution at room temperature without any special atmosphere (just atmospheric environment, 101 kPa). Thiourea and PEG(6000) were added to double-distilled water in turn (40 mL solution in a beaker of 50 mL), then a few drops of diluted sulfuric acid were added to make the solution acidic. After that, FeSO_4_**·**7H_2_O was added and then diluted sulfuric acid was again used to adjust the value of pH of the solution to an optimized value. Each step was accompanied by stirring to make the solution homogeneous. Thiourea was used as a sulfur source, and PEG was used to make the film flatter (note: PEG is a common electroplating additive in the electroplating industry, and it does not affect the film composition). We performed a series of optimization processes, including deposition potential, pH, sulfurization temperature, etc. Indium-tin oxide (ITO)-coated glasses were used as substrates for deposition. Prior to usage, the ITO was scrubbed with detergent and then ultrasonically cleaned by acetone, alcohol, and double-distilled water for 15 min, followed by a UV lamp cleaning for 20 min. The anode used in the electrolytic cell was a platinum (Pt) sheet and the reference electrode was Ag/AgCl with saturated potassium chloride (KCl) electrolyte. The preparation condition for the precursor film was 40 mL double-distilled water with 37.5 mM FeSO_4_, 262.5 mM thiourea, and 0.2 g/L PEG (6000) in it and pH = 3.3. The deposition potential varied from −1.0 V to −1.3V.

The precursor film fabricated by ECD was put into a tube furnace and annealed in a sulfur vapor atmosphere (i.e., sulfurization). The tube furnace had two temperature zones (i.e., Zone 1 and Zone 2): Zone 1 for heating sulfur powder, providing sulfur steam; Zone 2 for sulfurization of films. The distance between sulfur powder and precursor film, which were both placed on quartz glass, was about 20 cm. The sulfur vapor spread to the temperature zones with no carrier gas, and sufficient sulfur powder (0.450 g) was provided to ensure that there was still residual sulfur when finishing the sulfurization with pre-determined time. The tube furnace was evacuated to less than 1 Pa before heating. During heating, the vacuum pump was closed and the inside of the tube furnace was isolated from the outside. The heating temperature for sulfur powder was 180 °C, and the temperature for sulfurization was varied from 400–550 °C.

In device fabrication, P3HT film was prepared on iron pyrite film by spin-coating, where the solvent was chlorobenzene and the P3HT concentration was 15 mg/mL. After spin-coating at 4000 rpm for 28 s, the film was annealed on a hot plate at 100 °C for 3 min, resulting in a thickness of 70 nm. The silver electrode (about 100 nm) was prepared by thermal evaporation with a deposition rate of 0.4 Å/s.

X-ray diffraction (XRD) patterns were obtained using a Bruker diffractometer (D2 Phaser) with Cu K_α_ radiation (λ = 1.54184 Å). UV-visible absorption spectra were conducted on a Perkin-Elmer spectrometer (Lambda 750). The surface morphology was characterized using a field-emission scanning electron microscope (SEM) (FEI Helios G4 UC, Hillsboro, OR, USA). Electrochemical deposition, cyclic voltammetry and an impedance potential test were carried out with an electrochemical workstation (Corrtest CS2350H Bipotentiostat, Wuhan, China). Element compositions of the films were analyzed with energy-dispersive X-ray spectroscopy (EDX) (Bruker, Quantax Q80, Ettlingen, Germany). Raman spectra were taken by a Nano Finder 30A (Tokyo Instrument, Inc., Japan) equipped with a 532 nm laser. Thickness was measured with a profilometer (AlphaStep D-300, KLA Tencor, Ballston Spa, NY, USA). The current density vs. voltage (J-V) of heterojunction solar cells were measured under 100 mW/cm^2^ at AM 1.5 illumination (Newport Oriel Sol3A, Irvine, CA, USA).

## 3. Results and Discussion

**Figure 1:**
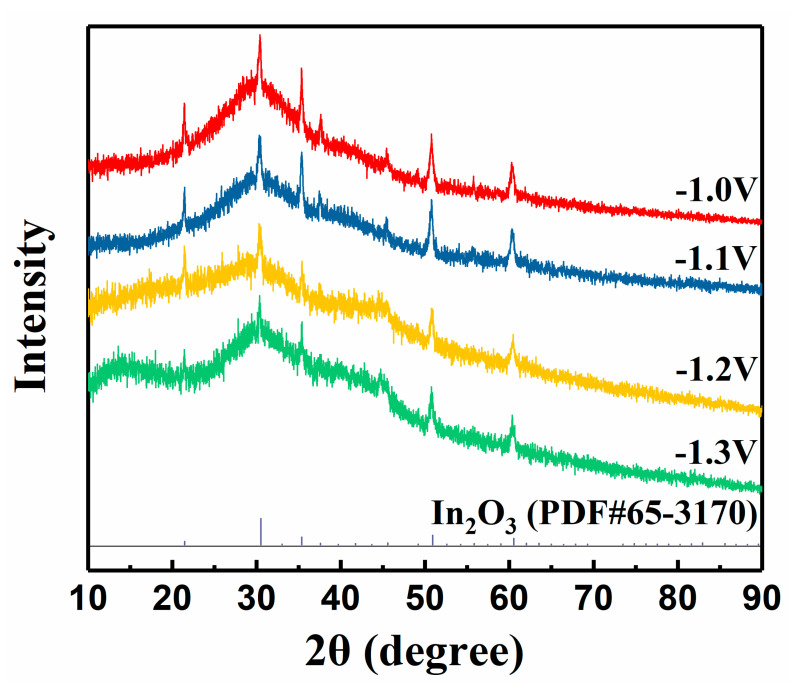
XRD patterns of precursor films deposited at different potentials.

First of all, we deposited films on ITO substrates and explored the effect of deposition potential on the crystallization of films. The XRD patterns of precursor films are shown in Figure 1, and only the diffraction peaks of the ITO substrates can be observed, indicating that the films are amorphous. As deposition potential increases from −1.0 to −1.3 V, the amorphous film becomes thicker because the substrate-ascribed peaks are suppressed. The amorphous nature is consistent with the report of R. Henriquez et al., and similar results also appear in reports using Na_2_S_2_O_3_ as a sulfur source [17,18,24]. Note that we found that there were bubbles on the sample and that they attached to the film when the deposition potential was larger than −1.1 V, which may be caused by the hydrogen evolution reaction of the electrode. Accordingly, we chose −1.0 V as the optimized deposition potential.

**Figure 2:**
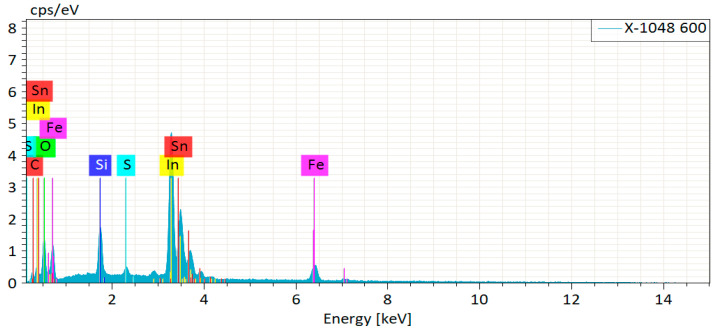
EDX result of the precursor film deposited at −1.0 V.

**Figure 3:**
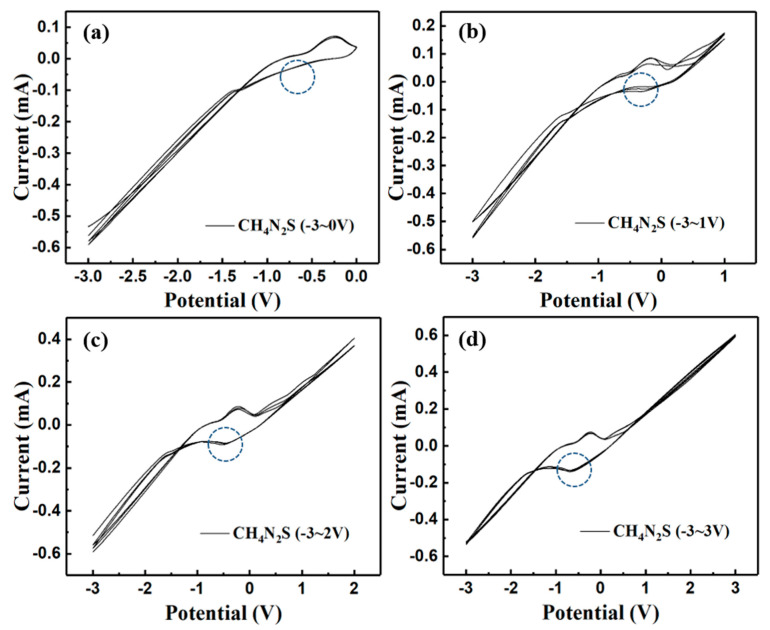
Cyclic voltammograms of Pt in thiourea aqueous solution at different scanning potential ranges of (**a**) -3~0 V, (**b**) -3~1 V, (**c**) -3~2 V, (**d**) -3~3 V.

In order to characterize the composition of the precursor film, we performed EDX measurements, and the results are shown in Figure 2 and Table 1. The oxygen element content is high, which may originate from the SiO_2_, In_2_O_3_, and SnO_2_ in the substrate. According to the ratio of sulfur and iron content, the precursor film is a compound with very low sulfur content, rather than 2:1 of sulfur and iron. Combining the XRD and EDX results, it can be inferred that the precursor film is an amorphous film containing iron and sulfur, rather than FeS_2_ film. In order to understand the specific reactions, we carried out a cyclic voltammetry test of Pt wire in an aqueous solution of thiourea with three cycles per scan. As shown in Figure 3a, when the scanning potential range is −3~0 V, there is no reduction peak. But when the potential range contains a positive potential, a reduction peak appears (Figure 3b). Moreover, as the positive potential range expands, the reduction peak becomes more and more obvious (Figure 3c,d).

The reduction peak does not correspond to the reduction reaction of thiourea, but the reduction of the product obtained from the anodic oxidation of thiourea. Such a result gives support to the view of Prabukanthan et al.’s report that thiourea first forms formamidine disulfide and then joins the reduction reaction (Equation (1)) [23]. Based on the fact that the precursor film is a sulfur-iron compound with low sulfur content, rather than FeS_2_ itself, we inferred the possible reactions to form our precursor film, as described in Equation (2).

2CH_4_N_2_S → [HN=C(NH_2_)S]_2_ + 2H^+^ + 2e^−^ (1)

Fe^2+^ + 2e^−^ + [HN=C(NH_2_)S]_2_ → FeS_1−x_ (2)

FeS_1−x_ + (1 + x)S = FeS_2_ (3)

**Figure 4:**
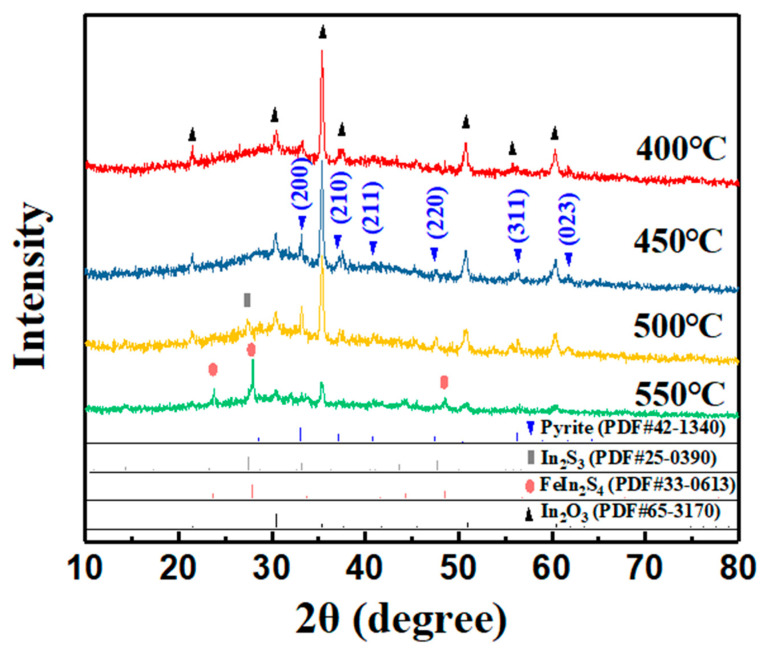
XRD patterns of films obtained by sulfurization at different temperatures (film thickness is about 200 nm).

In order to convert the amorphous precursor film into crystalized FeS_2_, we tried sulfurization as a post-deposition treatment (PDT) and studied the effect of sintering temperatures during sulfurization on the properties of the film. The XRD results of PDT-ed film at different sintering temperatures are shown in Figure 4. The FeS_2_ diffraction peak of (200) plane appears when the sulfurization temperature was 400 °C, and the peak becomes higher at 450 °C, indicating increased crystallinity and grain growth. The reaction during sulfurization is represented by Equation (3). The sintering at 500 °C further improves crystallinity; however, the ITO starts to decompose. Furthermore, the Bragg peak at 27.5° represents the appearance of the In_2_S_3_ phase. When the temperature further increased to 550 °C, the diffraction peak of FeIn_2_S_4_ appears due to the reaction between the film and the substrate. Consequently, 450 °C is the optimized sulfurization sintering temperature.

**Figure 5:**
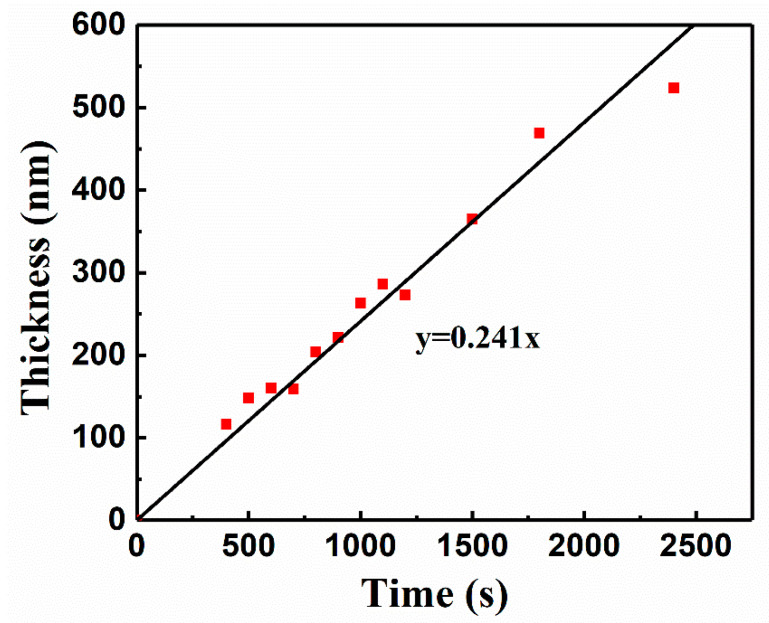
Variation of the thickness of FeS_2_ film as a function of electrochemical deposition time.

**Figure 6:**
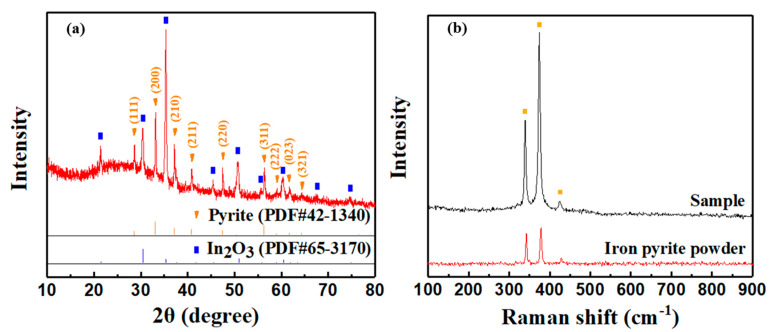
(**a**) XRD (film thickness is about 520 nm) and (**b**) Raman spectra of iron pyrite film. The Raman spectrum of pure iron pyrite powder is also given.

In Figure 4, we can also find that the XRD peak intensity of the film treated under 450 °C is relatively weak due to the relatively lower thickness (thickness = 200 nm). Then we explored the deposition rate of preparing FeS_2_ film. Figure 5 depicts the relationship between the thickness (*y*) of iron pyrite film (note: not precursor film) and the electrochemical deposition time (*x*). The thickness is linear with the deposition time, which follows the fitted formula *y* = 0.241*x*. We simply extended the deposition time and obtained thicker film of about 520 nm. The XRD result in Figure 6a illustrates that the film is a pure iron pyrite film with drastically enhanced diffraction peaks. Moreover, the ratio of peak intensities is consistent with JCPDS card No. 42-1340. In order to further confirm the purity of the film, we performed Raman measurements, as shown in Figure 6b. The spectra of the iron pyrite film are consistent with that of the purchased iron pyrite powder. Three Raman peaks at approximately 339 cm^−1^, 374 cm^−1^, and 426 cm^−1^ originate from iron pyrite and correspond to the S_2_ libration, in-phase stretch, and coupled libration/stretch vibrational modes, respectively [15,25,26,27], which demonstrates that it is a pure iron pyrite film. The results clearly manifest the advantage of sulfurization sintering for the preparation of FeS_2_ thin film as compared with the report without sulfurization in the ECD method [17,23]. Therefore, we believe sulfurization is a necessary process in building crystallized and pure iron pyrite film in the ECD method.

**Figure 7:**
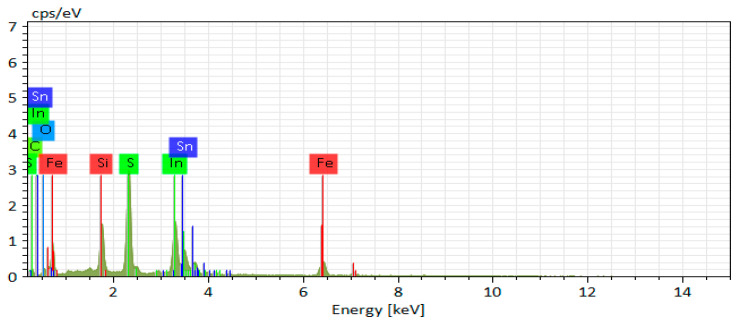
EDX result of iron pyrite film.

**Figure 8:**
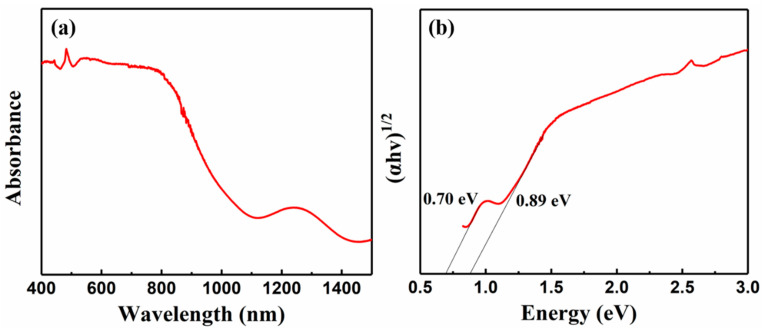
(**a**) Absorption spectrum and (**b**) Tauc plot of the iron pyrite film.

The composition of iron pyrite film was characterized by EDX measurements, and the results are shown in Figure 7 and Table 2. The atomic ratio of sulfur and iron in the film is 1.91:1, which means there are some sulfur vacancies in the film. The absorption measurement of iron pyrite film was carried out to determine the band gap of the film. As shown in Figure 8a, the film shows strong absorption in the range of 400–850 nm, suggesting a potential absorbing layer for solar cell in the visible region. The band gap of the film was calculated by a Tauc plot according to the (αhυ)^1/2^ v.s. hυ relation [13]. A sharp absorption edge at around 950 nm was observed, corresponding to a band gap of 0.89 eV, as shown in Figure 8b. There is also a small absorption edge around 1350 nm, corresponding to a band gap of 0.70 eV. The extra absorption edge and the relatively smaller band gap (0.70 eV) in the film may indicate some undetected impurities. Similar results have been reported by Srivastava et al. [28], and there are even two or more small band gaps in other reports [29,30]. However, we did not observe impurity in XRD and Raman. Therefore, the smaller band gap may be related to the sulfur vacancies in the film that were detected in EDX, which is similar to the guess of de las Heras, C. et al. [30]. The small band gap is not conducive to the photovoltaic application of iron pyrite, and undoubtedly it needs further research.

**Figure 9:**
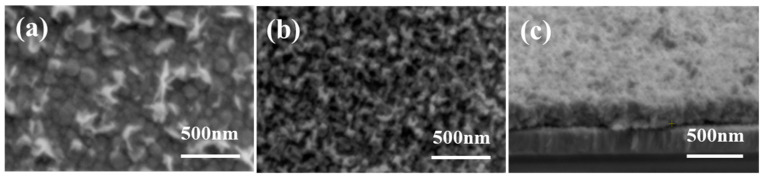
SEM images of (**a**) precursor film, (**b**) iron pyrite film, and (**c**) cross section of iron pyrite film on ITO.

The surface morphologies of the precursor film and iron pyrite film are shown in Figure 9a,b, respectively. The precursor film is not very continuous, with characteristics of two different phases. After sulfurization, the surface morphology of the film changes obviously, and the film becomes even and continuous. The cross section of the film is shown in Figure 9c. The iron pyrite film is flat and dense. The surface morphology of the film is comparable to that prepared by spin coating [15,29].

As we all know, the conductive type of absorber layer is of great importance to constructing a device. However, for the conductive type of FeS_2_ films, it is still a concern in recent years [31,32]. So far, the reported undoped iron pyrite thin films are typically p-type while most undoped iron pyrite single crystals are n-type [33]. There is also an unexpected phenomenon that an inversion layer leading to p-type conductive behavior was formed at the FeS_2_ film surface [15,34]. Most of the conductive types are measured by Hall measurements. However, when the film has a conductive substrate or low mobility, typical Hall measurements become inaccurate or even powerless [29]. Considering our iron pyrite film was coated on ITO substrate with good conductivity, the Mott–Schottky (MS) measurement may exert its own unique advantages to let us obtain the conductive type of our iron pyrite film [35]. Then we performed the Mott-Schottky test. The MS plot is described by Equation (4) and *C_SC_* is calculated according to Equation (5) [36,37]. We mark *k* as the slope of the curve in Equation (6), and Equation (6) can be simplified to Equation (7) after importing the values of *e* and *ε_0_*.

(4) $$\frac{1}{C_{SC}^{2}}\;=\;\frac{2}{e\epsilon \epsilon_{0}N_{A/D}}{(V-V}_{BP}-\frac{KT}{e})$$

*C_SC_* = −1/(2π*fZ*″) (5)

2/(*eεε*_0_*N*_*A*/*D*_) = *k* (6)

*N_A/D_* = 1.41 × 10^32^/(*εk*) (7)

*C_sc_* is the capacitance of the space-charge layer. *ε_o_* is the absolute permittivity of the vacuum (8.85 × 10^−14^ F**·**cm^−1^), *ε* is the dielectric constant of iron pyrite and *N_A/D_* is the effective concentration of electron or hole (cm^−3^). *e* is the charge of electron (1.6 × 10^−19^ C), *K* is Boltzmann constant (8.617 × 10^−5^ eV**·**K^−1^), *T* is temperature in Kelvin, *f* is frequency, *Z″* is the imaginary component of the impedance, and *V_BP_* is flat-band potential. The relation between *V* and *Z″* was characterized with an impedance potential test which we did at a frequency of 10^5^ Hz in an aqueous solution of sodium sulfate with 0.1 M. The working electrode, counter electrode, and reference electrode were iron pyrite sample, Pt wire and Ag/AgCl with saturated KCl solution, respectively.

**Figure 10:**
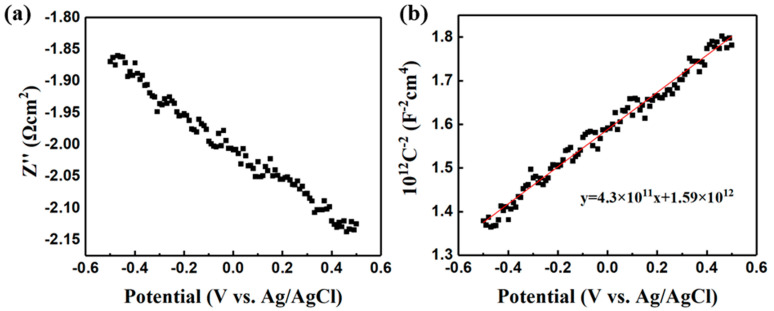
(**a**) Impedance potential plot of iron pyrite film in neutral solution (Na_2_SO_4_, 0.1 M), and (**b**) the Mott–Schottky plot of the iron pyrite film.

The impedance potential test result is shown in Figure 10a and the MS plot is shown in Figure 10b. It can be inferred that the conductive type of the film is n type because the slope of the MS curve is positive [37]. When *ε* = 10.9 F·cm^−1^ [38] and curve slope *k* = 4.3 × 10^11^ were used in Equation (7), the carrier concentration of *N_D_* = 3.01 × 10^19^ cm^−3^ was obtained. The flat-band potential *V_BP_* = −3.70 eV is also easy to be calculated according to the horizontal intercept (the value of *KT/e* is negligible because it is relatively too small) [37].

**Figure 11:**
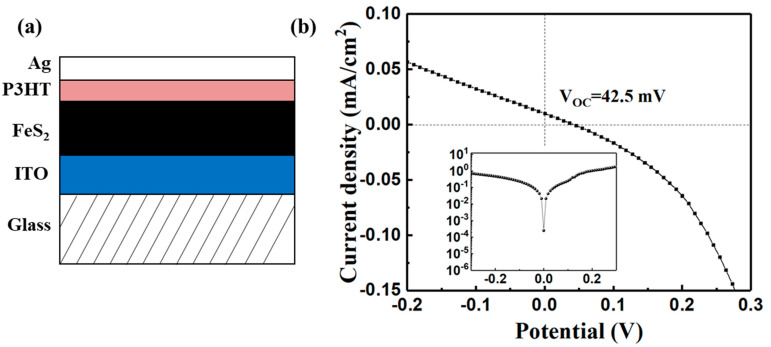
(**a**) Structure of FeS_2_/P3HT heterojunction thin-film solar cell, and (**b**) current density vs. voltage (J-V) characteristics (insert: dark J-V curve).

We characterized the photovoltaic response of the iron pyrite film in PV devices, and the structure of the device is shown in Figure 11a. The result is shown in Figure 11b. It shows that the device exhibits photovoltaic properties. The open circuit potential (V_OC_), short-circuit current (I_SC_), and fill factor (FF) of the device are 42.5 mV, 0.01 mA/cm^2^ and 25%, respectively. The dark J-V measurement (insert in Figure 11b) clearly indicated a rectification characteristic of the device. Although the device performance is not so attractive, the results confirm the photovoltaic effect of the FeS_2_ thin film. It also suggests that FeS_2_ is an n-type semiconductor because of the p-type nature of P3HT [39,40]. This is also consistent with the MS measurement result as discussed above.

## 4. Conclusions

We synthesized precursor films using thiourea in electrochemical deposition and then post-treated the films in a sulfur atmosphere (i.e., sulfurization) under different temperatures. The precursor films are amorphous and then turned into crystalline FeS_2_ films after sulfurization sintering. The optimized sulfurization temperature was 450 °C. The obtained FeS_2_ film was pure, conformal and smooth. The electronic characterization indicates that it is n-type with a carrier concentration of 3.01 × 10^19^ cm^−3^ and flat-band potential at −3.70 eV. The FeS_2_/P3HT heterojunction thin-film solar cell exhibits a response to light. This work suggests that sulfurization is very important in building crystallized and pure FeS_2_ films in electrochemical deposition. With further efforts, we are eager to further improve the device performance.

## Figures and Tables

**Table 1:** Atomic percentage of the precursor film deposited at −1.0 V.

Element	C	O	Si	S	Fe	In	Sn
Percentage	5.37	54.00	7.92	1.17	8.82	20.93	1.79

**Table 2:** Atomic percentage of iron pyrite film.

Element	C	O	Si	S	Fe	In	Sn
Percentage	27.82	14.60	9.07	21.89	11.45	13.57	1.60

## Data Availability

The data used to support the study are available from the corresponding author upon request.
